# [Retracted] Intestinal trefoil factor activates the PI3K/Akt signaling pathway to protect gastric mucosal epithelium from damage

**DOI:** 10.3892/ijo.2025.5759

**Published:** 2025-06-02

**Authors:** Zhaorui Sun, Hongmei Liu, Zhizhou Yang, Danbing Shao, Wei Zhang, Yi Ren, Baodi Sun, Jinfeng Lin, Min Xu, Shinan Nie

Int J Oncol 45: 1123-1132, 2014; DOI: 10.3892/ijo.2014.2527

Following the publication of the above paper, it was drawn to the Editor's attention by a concerned reader that, for the Transwell migration assay experiments shown in Fig. 1C and 3C, three sets of data panels were found to contain overlapping sections of data (including four panels across the figure parts that contained the same overlapping data). A further pair of overlapping data panels were identified for the immunofluorescence experiments shown in Fig. 5. Upon receiving notification of this issue, the authors requested a corrigendum and submitted corrected versions of Figs. 1, 3 and 5. However, upon performing an independent analysis of the data in this paper in the Editorial Office, it was also noted that certain of the data in these figures had appeared subsequently in a pair of more recent publications by the same research group, and moreover, one of these images had been incorporated into one of the three revised figures submitted for the corrigendum.

Owing to the large number of duplications of data identified in the published paper, and the apparent mislabelling of the authors' own data files, the Editor of *International Journal of Oncology* has decided that it should be retracted from the Journal on account of a lack of confidence in the presented data. After contacting the authors, they accepted the decision to retract the paper. The Editor apologizes to the readership for any inconvenience caused.

